# From “*It Has Stopped Our Lives*” to “*Spending More Time Together Has Strengthened Bonds”*: The Varied Experiences of Australian Families During COVID-19

**DOI:** 10.3389/fpsyg.2020.588667

**Published:** 2020-10-20

**Authors:** Subhadra Evans, Antonina Mikocka-Walus, Anna Klas, Lisa Olive, Emma Sciberras, Gery Karantzas, Elizabeth M. Westrupp

**Affiliations:** ^1^School of Psychology, Deakin University, Geelong, VIC, Australia; ^2^Faculty of Health, Centre for Social and Early Emotional Development, Deakin University, Geelong, VIC, Australia; ^3^Institute for Innovation in Mental and Physical Health and Clinical Translation (IMPACT), School of Medicine, Deakin University, Geelong, VIC, Australia; ^4^Murdoch Children’s Research Institute, Melbourne, VIC, Australia; ^5^The Judith Lumley Centre, La Trobe University, Bundoora, VIC, Australia

**Keywords:** COVID-19, qualitative study, family relationships, Australia, social restrictions

## Abstract

The present study uses a qualitative approach to understand the impact of COVID-19 on family life. Australian parents of children aged 0–18 years were recruited via social media between April 8 and April 28, 2020, when Australians were experiencing social distancing/isolation measures for the first time. As part of a larger survey, participants were asked to respond via an open-ended question about how COVID-19 had impacted their family. A total of 2,130 parents were included and represented a diverse range of family backgrounds. Inductive template thematic analysis was used to understand patterns of meaning across the texts. Six themes were derived from the data, including “Boredom, depression and suicide: A spectrum of emotion,” “Families are missing the things that keep them healthy,” “Changing family relationships: The push pull of intimacy,” “The unprecedented demands of parenthood,” “The unequal burden of COVID-19,” and “Holding on to positivity.” Overall, the findings demonstrated a breadth of responses. Messages around loss and challenge were predominant, with many families reporting mental health difficulties and strained family relationships. However, not all families were negatively impacted by the restrictions, with some families reporting positive benefits and meaning, including opportunities for strengthening relationships, finding new hobbies, and developing positive characteristics such as appreciation, gratitude, and tolerance.

## Introduction

The COVID-19 pandemic has resulted in unprecedented shutdowns, shortages, and sources of stress for individuals and families across the world. The Australian federal and state governments introduced an increasingly strict regime of social distancing/isolation measures to slow the rate of infection early in the pandemic (March to April 2020). These measures present significant risks to the population, over and above the health threat associated with COVID-19, including compromised family mental health and relationships (Holmes et al., 2020). Worldwide, the rate of mental health symptoms in adults at the time of the COVID-19 pandemic has been elevated compared to historical norms (Nelson et al., 2020). In addition to experiencing physical and mental health burden associated with COVID-19, Australians were in the unfortunate position of already undergoing significant hardship, having experienced devastating bushfires and floods directly before the pandemic. It is therefore important to understand the specific responses of Australian families, given that their psychological and financial resources may have already been compromised before the pandemic’s emergence.

Parents and children living through COVID-19 are faced with numerous challenges, which together present a constellation of risk. Recent research has identified that the top stressors faced by parents and carers during COVID-19 include work, their children’s well-being, and the well-being of family and friends outside their household (Waite and Creswell, 2020). Of concern, two thirds of parents report that they are not meeting the dueling needs of work and their child’s well-being (Waite and Creswell, 2020). Other likely concerns include high rates of unemployment and economic uncertainty, a reduction in social support and onsite schooling, and a reduction in access to critical clinical, community and sporting activities, including greenspace and playgrounds. Each of these challenges is known to negatively impact family well-being outside the context of a pandemic (Marin et al., 2011; Olesen et al., 2013; Bakusic et al., 2017; Leigh-Hunt et al., 2017; Wang et al., 2017; Lupien et al., 2018). Now co-occurring together during an unprecedented health crisis, these conditions may be pushing families to the edge of their resources.

Prior research documenting the effect of natural and man-made disasters provides clues as to the extent of the challenges faced by families during the COVID-19 pandemic. Mental health problems, domestic violence and family conflict are common sequelae to natural and man-made disasters (Reifels et al., 2019). For example, rates of family conflict and domestic violence increased during and in the aftermath of crisis events, such as the 2010–2011 Canterbury earthquakes in New Zealand and the 2009 Victorian Bushfires in Australia. The COVID-19 pandemic also presents other unique challenges. Being “locked in” with family members, while parents juggle paid work and supervise home-schooled children for extensive periods of time is an entirely new and unstudied phenomenon in modern history. Dealing with work and family responsibilities is already known to stress families, leading to parent mental health problems, couple conflict, and child mental health problems (Westrupp et al., 2015; Dinh et al., 2017; Vahedi et al., 2019). Balancing work and family along with responsibility for children’s education, while in constant close physical proximity to partners and children, presents a highly unusual situation.

Given the unprecedented demands placed on families during COVID-19, qualitative work is necessary to explore the myriad impacts upon family life, and importantly, to give voice to those directly impacted. Such an approach allows for the inclusion of unexpected findings and a broad range of experiences. Remaining open to the diversity of experience, allowing responses that may span from tragedy and risk to benefit finding, not only captures the many ways that humans adapt to challenge, but also better supports appropriate and nuanced intervention in such circumstances. It is possible that the pandemic crisis will have a negative impact on families in general, but particularly on vulnerable parents and families, while those families without pre-existing risk factors may report minimal disruption or even thrive. Indeed, qualitative research with individuals living through natural disasters has triangulated quantitative data showing common concerns of worry over finances and mental health, but also that some individuals report post trauma growth (Rowney et al., 2014). Employing an inductive approach, which allows the data to speak for themselves, the present study therefore aimed to explore family experiences in response to the early stages of Australia’s COVID-19 social distancing/isolation restrictions.

## Materials and Methods

### Design

This cohort was part of a large longitudinal survey examining the impact of the COVID-19 crisis on Australian parents and children. The present study is focused on the qualitative data obtained during the first wave of the study. The qualitative data involved asking parents to describe the impact of COVID-19 on their families during the initial stage of lockdown in Australia, which began on March 23, 2020. The study was approved by the Deakin University Human Ethics Advisory Group (HEAG-H 52_2020).

A critical realist approach was taken, which argues that research is not independent of the researchers’ perspective but that there is a reality to observe and describe (Sims-Schouten et al., 2007; Braun and Clarke, 2013). Within this approach, a descriptive theoretical framework was employed to understand the experiences of families. The goal of descriptive qualitative studies is to provide “a comprehensive summary of events in the everyday terms of those events” and to take participant responses as they are rather than interpreting the data beyond what is provided by participants (Sandelowski, 2000; Kim et al., 2017). As such, researchers stay close to the data they are analyzing, and to the surface of words and experiences. From a practical perspective, this approach was selected as it was deemed an appropriate match to the “thin” nature of the data to be collected, where parents who were likely stressed working and caring for children, were asked a brief question at the end of a larger survey. Further still, it was reasoned that a comprehensive summary of these parental experiences was required given that COVID-19 was a one of kind event. Therefore, the approach was experiential, in that participants’ interpretations, experiences, and meanings were prioritized over *a priori* theoretical frameworks or even researchers own interpretations of the event. This is in contrast to a critical approach where the researchers’ interpretations are paramount (Braun and Clarke, 2013). The data for the present study were derived from a single open ended qualitative question that required a short-answer text response: “How has COVID-19 affected your family life?” Given little research has examined the impact of COVID-19 on families and the unique nature of the COVID-19 pandemic itself, an inductive analysis approach was taken, where we allowed the data to guide the identification of distinct patterns.

The final qualitative dataset contained 2,130 responses, with an average of 19 words per response (*M* = 19.78, *SD* = 24.80, range = 0–308). While examining short-answer text responses can result in a “shallow” qualitative dataset, we argue that in the case of this study, a “rich” dataset was obtained due to the large sample size (>2,000). Large samples in qualitative research increase the likelihood that a range of unique perceptions are obtained, thus enabling us to provide a wide-lens view of how Australian families experienced COVID-19. Critical realist approaches also assume that studies with large samples provide a representative picture of the population under study, especially for descriptive qualitative analyses (Sandelowski, 2000; Boddy, 2016). Additionally, analyzing a standardized open-ended qualitative question within a survey is suitable for when attempting to identify consistent patterns (Braun and Clarke, 2013).

In terms of reflexivity, which actively acknowledges how the researchers’ positions within the project contributed to data interpretation (Berger, 2015), all authors reside in Australia, thereby experiencing the same social distancing/isolation restrictions as participants. Those who read, coded, and interpreted the qualitative dataset specifically included both parents (SE, EW) and non-parents (AM-W, LO, AK), thereby ensuring that both insider and outsider perspectives were used to understand the data. The research team also contained expertise across a variety of psychology disciplines, including developmental (SE, ES, GK, ES), social (AK, GK), clinical (ES, EW, LO), and health (SE, AM-W, LO), thus helping to identify the multitude of ways in which the data represented psychological processes and phenomenon.

#### Recruitment

Families were recruited via social media advertisements and paid online recruitment platforms (e.g., Facebook, Reddit, Prolific). A range of methods have been used to target specific groups to increase the representativeness of the sample (e.g., targeting postcodes and demographic factors). The data from the present study were derived from the first wave of data, which occurred between 8 April and 28 April, matching the early period of the COVID-19 pandemic in Australia. This was a time when families were trying to navigate social restrictions/isolation restrictions and lockdown measures during the early stages of the pandemic. In particular the threat of infection was unknown, an economic downturn was threatening financial stability and employability, and families were facing new or emerging directives about home learning.

#### Participants

Participants were eligible to participate in the current study if they resided in Australia and spoke English, were 18 years or over, and a current parent of a child aged 0-18 years. Participants were under no obligation to participate and free to withdraw from the study at any time without consequences. A total of 2,130 parents who had completed demographic information and responded to the qualitative open-ended qualitative question were included in the present study.

### Data Analysis

Template thematic analysis was used to understand patterns of meaning across the texts (Brooks et al., 2015). The technique views text as a form of data and focuses on how each theme obtained from the text data can shed light on the topic of interest in an in-depth manner. This approach to data also views frequencies and other numeric data as unable to provide rich and meaningful insight into the text, unlike summative themes (King, 2012). Template thematic analysis attempts to balance structure and flexibility, using a high degree of structure in analyzing textual analysis in a way that can accommodate the individual requirements of research questions and data (King, 2012). Given the large dataset, and the number of researchers involved in analysis, we deemed it appropriate to employ a highly structured approach that was still suited to understanding meaning within the text. There are similarities to other popular ways of conducting thematic analysis (e.g., reflexive thematic analysis) (Braun and Clarke, 2019) and other qualitative approaches that involve templates. However, this technique allows for structure early in the analysis, including the development of a coding book before detailed analysis and the development of early themes. It also allows for a unique continual iterative development of the template, to support a coding process that captures all text relating to the research question.

Template thematic analysis involves a series of six steps including: (1) familiarization with the data through re-reading the transcripts – SE and AK read all transcripts, and 4 members of the analysis team each read one quarter of the data (AM-W, LO, AK, EW); (2) preliminary hand coding by the first author; (3) the organization of emerging themes into clusters; (4) the development of a coding template; (5) the iterative modification of the coding template, which was achieved by the analysis team each hand coding a distinct 10% of the data; this coding by the members of the research team resulted in changes to the coding names and template until a final template was produced that was able to capture all relevant sections of the text that was relevant to the research question; consistent with template analysis, additional codes were created by splitting existing codes and creating new ones; 6) and the finalization of the template to code the entire dataset. The entire dataset was then hand coded in excel by a coding team of 4 postgraduate students, supervised and trained in the approach and the template by the first author. Double coding was then undertaken after the entire dataset had been coded, with between 10% and 50% of the data double coded (SE, AM-W, AK), with any discrepancies discussed and resolved. The final list of themes was generated through discussion with the entire research team, which involved authors reviewing and discussing the coded transcripts and collaboratively determined themes and subthemes for analysis.

## Results

Demographic information, describing the participants is presented in Table 1. As shown, families represented a wide range of family make-up, parent and child sex and age, education, geographical region, and relationship status.

**TABLE 1:** Sample characteristics for the analysis sample of the COVID-19 Pandemic Adjustment Survey (CPAS) (*N* = 2,130).

Characteristic	*N* (%)
Parent age, m(sd)	38.4 (7.1)
Child age, m(sd)	8.6 (5.2)
**Parent gender**	
Cisgender men	398(19%)
Cisgender women	1,672(81%)
Transgender or non-binary	1(0.1%)
**Child gender**	
Cisgender boy	1,086(51%)
Cisgender girl	1,031(49%)
Transgender or non-binary	9(0.4%)
**Geographic location**	
Major cities of Australia	1,267(60%)
Inner Regional Australia	610(29%)
Outer Regional Australia	201(10%)
Remote Australia	38(2%)
**Number of children**	
1 child	613(29%)
2 children	979(46%)
3 children	389(18%)
4 or more children	148(7%)
Aboriginal or Torres Strait Islander	44(2%)
Language other than English	94(4%)
Parent born overseas	380(18%)
Low household income (<$52,000 per year)	298(14%)
Receiving government benefit	123(6%)
Single parent household	239(11%)
Did not complete high school	197(9%)
**Highest qualification**	
Trade certificate, diploma, or apprenticeship	502(24%)
University	1,467(69%)
**Unemployment**	
One parent unemployed	435(23%)
Two parents unemployed	34(2%)

*m(sd) = Mean (standard deviation).*

### Themes

The current study aimed to explore family experiences in response to the early stages of Australia’s COVID-19 social distancing/isolation restrictions. Employing a descriptive, inductive approach to the textual data obtained, we derived six themes using template analysis. These themes provided a comprehensive textual summary of how the COVID-19 pandemic impacted family life in Australia at one time point during the crisis (March 2020). Key findings and supporting quotes for each theme are summarized in the following text. Participants are identified with demographic information to demonstrate the diversity of voices within the sample, and to contextualize participants’ responses.

#### Theme 1: Boredom, Depression, and Suicide: A Spectrum of Emotion

For some families, restrictions weighed heavily, blanketing families in stress, and impacting multiple areas of family life. Parents used a variety of expressive adjectives to indicate the toll, noting their family was affected “heavily,” “massively,” and the experience was “chaos.” Other parents reported “it has stopped our lives” and “my family life has pretty much gone to shit.” For one family, the pandemic resulted in a cascade of stress that felt so unbearable that suicide had been considered. This parent’s response demonstrates the limitations in government support during the pandemic, such as mental health and financial resources. While this finding did not apply to all individuals equally, it was clear there was a heavy burden of financial loss for many families.

*It has put extreme stress on us with our 3 businesses closing, we are eligible for none of the gov. support due to a tax debt and are looking at bankruptcy and selling our home as the only option. Both of us have had thoughts of suicide.* Father of 3 children.

Many families reported a concern over deteriorating mental health in themselves, their children, and their families. Sometimes this appeared to be related to an exacerbation of existing mental health difficulties, including symptoms of depression, anxiety, and post-traumatic stress. Such findings illustrate the need to consider theory related to diathesis-stress, which identifies that mental health issues arise due to an interaction between predispositional vulnerabilities and precipitating stressors (Patten, 2013). As such, parents and children with vulnerabilities may require early preventative support to ensure that crises such as COVID-19 do not form the precipitating stressor leading to the emergence or worsening of mental health problems.

*It’s affected my child greatly. She is diagnosed anxiety disorder and is very stressed over the lockdown.* Mother of 1 child.

For one mother of 3 children who had recently experienced post-partum depression, the lockdown prevented her from accessing the care she needed to support her well-being, as she could no longer “implement any of the self-care or coping strategies we had in place in terms of outside support for me during this time,” which included catching up on rest and sleep while the children were usually at school. It is important to take note of such responses in developing mental health policy, as it is clear that many parents knew what they needed to support their mental health, but they were unable to access the measures so imperative to their mental health.

In other cases, it appeared that the pandemic was associated with new mental health problems in children and parents, including reports of being more paranoid in public and the emergence of mental health concerns.

*The children’s mental health has significantly suffered. 7-year-old cries herself to sleep most nights and 5-year-old has become very aggressive toward her sisters and Me*. Mother of 3 children.

Parents were particularly concerned about the mental health and welfare of their children, who seemed to struggle to understand this “frightening” new world. Such responses are concerning given knowledge that early childhood mental health issues are associated with later episodes of depression (Luby et al., 2014). In future crises, governments should act promptly to ensure that children displaying symptoms of worry, fear and sadness are offered prompt and age-appropriate mental health support.

*It’s created a lot more pressure on the family, the children have all become scared to leave the house and touch things for fear of dying, they’re having nightmares, worried uncles, aunts and grandparents will catch it and die, they have trouble understanding why we can’t leave the house and all the new rules in place.* Mother of 3 children.

Comments revealed the inter-relationships between individual family members’ mental health, and the presence of emotional contagion, where anxiety and depression in one family member reverberated throughout the family. Such evidence of emotional contagion suggests that efforts to bolster mental health in one family member may be beneficial for the entire family.

*Feeling isolated and stuck in the house has increased my partner’s anxiety and depression, which in turn has affected my mental health.* Father of 1 child.

Consistent with the general finding of emotional contagion, some parents commented on the relationship between their own mental health and their child’s functioning:

*My mental health has taken a really bad hit and I’m struggling to support my children.* Mother of 2 children.

Another aspect of functioning that impacted mental health was disrupted sleep. Some parents reported that their or their child’s sleep had deteriorated, with a likely impact on mental health.

*Struggle with 2 schoolkids and 2 toddlers, too much work, too exhausted, stressed, mentally draining, physically exhausting, sometimes I get depressed, worried if me or partner might catch the virus, can’t sleep at night.* Mother of 4 children.

Most commonly, parents reported an array of difficult but perhaps more manageable negative emotions and feelings. Words used to describe how their families felt included “stressed,” “agitated,” “frustrated,” “worried,” “unsettled” and “pressured.” Feeling trapped was also common, which one parent described as “cabin fever syndrome.” Others reported their children were “sick of being locked up,” and “Immensely we want to leave the house.”

*Six people trapped inside a house, slowly loosing (sic) their minds.* Father of 4 children.

The experience of feeling trapped may be particularly stressful for Australian families, many of whom are used to enjoying an expansive country, and a culture of relative freedom in movement and outdoor recreation.

On the less severe end of the spectrum, families reported being “bored.” In some families, such boredom was associated with negative impacts: “We’re all getting bored so therefore cranky,” while in others, boredom was offset by the capacity for self-entertainment:

*We are all stuck at home together and the kids are a bit bored but they’re going enough to be easily entertained most of the time*. Mother of 2 children.

Family boredom tended to carry undertones of longing for usual life.

*Boredom, everyone on edge because there are no outlets anymore*. Father of 2 children.

#### Theme 2: Families Are Missing the Things That Keep Them Healthy

Parents overwhelmingly reported how being “stuck” or “trapped” at home resulted in their family “losing” or “missing out” on their usual activities, events, and strategies, which had helped to maintain the family’s “structure,” “well-being,” and “happiness.” Several parents discussed loss in both implicit and explicit terms. For instance, parents used words such as “missing,” “reduced,” “disrupted,” and even “non-existent” to describe the ways in which their family life had changed. Alternatively, parents listed these activities and strategies.

*Strained. Children stressed, missing friends, freedom, sporting activities, extended family, camping adventures*. Mother of 3 children.

Parents spent considerable space specifying the exact activities, strategies, or events that were now missing from the family’s day-to-day life. These were across a wide spectrum and varied from the practical, including access to “medical care” and “help,” to the more personal, including “social connection,” “physical touch,” and “exercising.” One cluster of parents talked about significant disruptions in their family daily routine, including shopping, schooling, and cooking, which was often stressful.

*More stress, disrupted routines, poor sleep, having to eat my cooking (I suck at cooking) and far less access to medical support for my disabled husband who was eventually admitted to hospital for lithium toxicity*. Mother of 1 child.

Parents spoke of the cancelation of both “normal” and “special” extra-curricular activities and events. These included events that the family did together, such as attending church, going on holiday, and engaging in family activities such as hiking and camping, and frequenting museums and playgrounds. There was also discussion of activities that were separate from the family, including exercising, playing sports and attending playdates. Some parents also spoke of loss over the cancelation of important events such as funerals, birthdays, and weddings. Overwhelmingly, the loss of activities outside the home was difficult for families to reconcile, as such activities were integral to family well-being and hope.

*We used to see family, friends, go to church and do kids activities like playgroup a lot—We had at least one activity or visit per day of the week. Cutting all of that out to stay home has been hard. We miss being able to see our family and friends, to do activities outside of home that are more than a walk around the block. We’re all tense and exhausted.* Mother of 3 children.

*Stuck at home, missed birthday parties, will miss out on great nanas funeral, kids missed visiting great nana for the last time before she passed away (not from corona).* Mother of 3 children.

Although sport, church, playgroups and family gatherings may be considered extra-circular, such activities and events appear to be essential to well-being. This is unsurprising, given Australians have a strong culture of engaging in extra-curricular activities, including sport. Such activities not only help family members to bond through shared experiences, but also help families to build social connections to individuals and communities outside their family unit.

Finally, there was a loss of social connection, especially in relation to external family and friends. This lack of social connection appeared to partially explain the negative emotions, and stress reflected within Theme 1. Many parents spoke of how family members were “missing” seeing others, and how the reduction in usual social support networks were placing additional strain on the immediate and extended family.

*The biggest issue is the separation from my 70-year-old (diabetic) mother who until March had never been apart from my daughter for more than a week. She is widowed and I worry the risk of loneliness is worse than that of corona.* Mother of 1 child.

*The biggest affect has been the cancelation of all sporting & activities for our kids & the need to be at home & to continue schoolwork at home. They are also feeling isolated socially due to being very social kids & not having social media to interact with friends.* Mother of 3 children.

Many parents reported that they and their family members were using online communication to stay in touch with friends and family, but many also commented that this mode of communication was “not the same” and did not replace in person contact. This distinction between natural, in-person connection and connection through technology is an important comment on the inferiority of technology to replace human interaction, given the worldwide focus on using technology to stay connected and healthy during the pandemic. It seems the digital space is a necessary but poor replacement, particularly for children who are still developing their social skills.

*We are all in the house all the time. Kids used to do sport 6 days a week now none. The children are struggling to stay connected with their friends as video chat is not the same as hanging out.* Mother of 2 children.

Although there was a strong emphasis on the negative consequences of loss, some parents acknowledged that the social isolation and restrictions had resulted in unexpected benefits for the immediate family, including more quality time together, as explored in greater detail in Theme 3.

#### Theme 3: Changing Family Relationships: The Push Pull of Intimacy

Many families indicated that the restrictions had stressed their relationships. Responses varied in severity, with some parents commenting that separation or divorce was a likely outcome of being housebound with their partner. Comments regarding separation may reflect the additional pressure placed on the couple relationship in the context of social distancing and enforced time spent together, or may have been initiated by the stress of the restrictions. For some of these families, existing relationship difficulties were exacerbated by pandemic-related stressors. For others, the impact of the pandemic seemed to be more complicated, with stressors heightening existing vulnerabilities in parents themselves (i.e., mental health problems), which made it harder to effectively maintain relationship satisfaction.

*It’s made me realize that my “marriage” is over. Work (for him) will always come first.* Mother of 1 child.

*We are getting divorced.* Mother of 2 children.

Although most families did not express this degree of relationship distress, strained family relationships were common, including increased conflict and arguments between parents, parents and children, and between siblings. Children in particular seemed to be a source of family relationship discord, either demanding more from already exhausted parents, or creating tension in the family by bickering and fighting as a result of being “cooped up”:

*We have too much time together. We are often irritable with each other. My child wants more social interaction from me that I can’t give.* Mother of 2 children.

*Being together all the time can be draining & our girls get on each other’s nerves. And ours.* Mother of 2 children.

For many, there was a sense that goodwill between family members was “wearing thin,” providing an indication that families can tolerate a certain amount of enforced time together, but there are limits, especially in families where relationships may already be strained. There was also further evidence that partners impacted each other:

*Too much time at home sucks. Tension between me and my partner over different thoughts about the pandemic ave (sic) restrictions in place.* Father of 4 children.

Other parents commented on not having enough time together, since they were constantly attending to children, with no capacity to take a break from the parent role.

*My husband and I get less chance for time together without the baby which we are starting to feel*. Mother of 1 child.

In other situations, couples had reduced contact due to their partner’s work, such as healthcare workers who kept their distance from family members at home, when a parent was “stuck” overseas, or had increased their hours at work. Relationships were also more difficult for separated family members with shared custody, and some children were not able to access parents who lived across state borders. Underlying many parents’ comments was the sense that over time, families had developed effective techniques for coping with normative life, family and work responsibilities, but the pandemic had wreaked havoc on these systems, and many were struggling to find a way through this new tangle of stress and shifting norms.

However, this was not the case for all parents, with one father commenting that his co-parenting situation had improved:

*I am a single parent. If anything it has increased the amount of friendly and random conversations with my ex-wife. Even had her over for Good Friday dinner.* Father of 1 child.

Indeed, for a number of parents, the pandemic resulted in closer bonds between family members, who were more engaged and connected to each other than ever before.

*It’s been great. Lots of quality time together.* Father of 3 children.

*Self-isolation* = *LOTS of time playing with the kids.* Father of 2 children.

A number of mothers also identified positive outcomes from having everyone at home together.

*We have spent more time together, slowed down, become more mindful.* Mother of 1 child.

*It’s actually improved our relationships as we have time to work through issues.* Mother of 3 children.

However, many parents noted conflicted feelings. There was a sense that the situation allowed more time and opportunity to develop relationships, but this benefit came with associated costs to personal freedom and space.

*Brought us closer but also more stressed.* Mother of 2 children.

*Lots of quality time. Lots of non-quality time.* Mother of 2 children.

In these families, it was evident that although the pandemic brought stress and conflict, there was also an opportunity for growth in family, couple and sibling connections. Some families were able to develop shared interests, and bond over new activities and hobbies:

*We have been arguing more as we are together 24/7. However we are playing more games together and trying to find interesting things to do.* Mother of 1 child.

In the families noting conflicted or mixed emotions, introspection seemed to be key in dealing with the challenges to forge stronger family bonds:

*During this time, as our house is small, we all went a little stir crazy; it forced me to be more honest about my emotions with my child; and made me utilize some parenting strategies (involving screen time) that I’d previously avoided. The uncertainty is creating more tension in the house than before; but we are all being honest about our feelings and trying to help each other understand how we feel.* Mother of 1 child.

#### Theme 4: The Unprecedented Demands of Parenthood

Parents reported being busier than usual, sharing examples of increased household chores and family management tasks, ranging from more frequent trips to the supermarket due to restrictions and not being able to find common household products, to increased cleaning needs with the entire family at home. Extra duties were perceived as difficult to manage, and resulted in an impact on physical health for some:

*My De Quervain’s in my right forearm has flared up and the same is developing on my left due to increased home duties and more time with children*. Father of 2 children.

Families particularly missed the support of friends and family who helped with childcare and chores. This was apparent in families where both parents worked:

*Reduced availability for work as my mother can no longer babysit and so we’re all juggling caring and work responsibilities which add stress.* Mother of 1 child.

Single parents and those with young babies felt the withdrawal of family support. The strain on single parents, who may be working full time, and under immense pressure to perform well in their job if they are the only provider, as well as taking sole responsibility for children at home should be underscored.

*We no longer see other family members, including the children’s father. I no longer have access to my support networks as a single parent*. Mother of 3 children.

The overarching message relayed by these busy families was that the requirement to do more than ever was straining their resources to cope. Parents described a difficult balancing act, of having to “juggle” multiple, competing roles to fill the vacuum left by their family’s usual care, education, and healthcare providers. The loss of important structures in the community, particularly schools, reveals the extent to which such institutions play a pivotal role in raising healthy families and children, with parents alone unable to provide the village that children need.

*Covid-19 had turned me into a stay at home mum, primary teacher, speech therapist, occupational therapist, strict budgeter, with no social outlet or relief. And I’m doing this alone with my healthcare worker husband being overworked.* Mother of 3 children.

*Stressful. I am the worker, the mother, the teacher, the cleaner, the cook, the shopper, the counselor, the sports coach, the therapy assistant.* 48-year-old mother of 2 children.

Balancing work or study with home schooling was burdensome and beyond the capabilities of many parents. Some reported having to work late into the night in order to accommodate the demands of employment and home schooling, potentially impacting their sleep and mental health. In fact, the difficulties navigating this unprecedented balancing act offered clues as to why parents reported stress and challenges to their mental health:

*The care load combined with paid work load is completely unmanageable. We are all very stressed, tired and cranky.* Mother of 2 children.

In addition to this difficult balance, an exacerbation of normal parent concerns was rife, including worries over children’s health and development. Parents expressed concerns about their children’s emotional, social, and cognitive developments that were heightened due to the isolation. In particular, parents were worried about their child “missing education” and missing out on the opportunity to develop social skills:

*All our other socialization activities have been canceled (playgroup, storytime at the library, visiting our local toy library). They can’t see their friends or cousins and I feel that it will impact on their social skill development- particularly for my eldest, who is autistic.* Mother of 3 children.

Parents were also concerned about their children’s growing use of screen time, with many feeling that children were relying too heavily upon screens for education and recreation. The extent of screen time to access normal activities was discussed by one mother.

*There’s a LOT more screen time than before the pandemic, given all the activities for kids are online, plus video calls to extended family, church is online now, and other things like live-streaming of animals from zoos etc.* Mother of 2 children.

There were also new and unexpected concerns that seemed beyond the remit of normal parenthood. For example, some parents were worried about their child contracting a deadly virus, that their children were now stressed by physical contact with family, including hugs, and were worried when their parents had to work outside the home. For parents employed in healthcare, or other high risk environments, there was additional concern:

*I work in health care and we are worried about me returning to work due to infant immunity*. Mother of 1 child.

Overall, there was a sense of fear for the ongoing well-being of family life*:*

*It has made us scared for our health and future and has isolated us from society and family*. Mother of 3 children.

Other work related demands involved having to work longer and harder, taking on multiple jobs, and bringing confidential or sensitive work home that was deemed problematic in terms of exposing children to inappropriate information. This is an important consideration, given that much has been made of the benefits to families who are now able to work from home. Working from home may result in unique stresses in some families, or even risk to children’s welfare. Moreover, an established body of evidence shows that the conflict inherent in parents juggling work and family demands has detrimental impacts on parent mental health, the couple relationship, and children’s long-term mental health (Dinh et al., 2017; Vahedi et al., 2019).

*I have to do my highly sensitive work from home a number of days per week. The risk of vicarious trauma to my family is significant in overhearing some of the conversations I am involved with*. Mother of 1 child.

#### Theme 5: The Unequal Burden of COVID-19

It was clear that some family characteristics and situations made the restrictions especially difficult to endure, or presented unique challenges. In particular, families of children with disabilities or physical or mental health conditions seemed to experience heightened stress.

*Our eldest is on the autism spectrum, so the impact on her is enormous, which then creates an impact on the family. This is especially compounded because there is no way to get time away from her, so we are all on high alert all the time.* Mother of 3 children.

*We were approaching a critical appointment for our youngest that may have ASD - ADD - ADHD but it has been canceled. Being stuck in the house with a wild child and unable to get relief. Not being able to shop with a child that touches everything*. Mother of 3 children.

Similarly, mental health difficulties or atypical neurodevelopment in parents presented challenges to the functioning of the entire family. Again, it appeared that enduring vulnerabilities in parents made the management of stress and relationships all the more difficult.

*Oh, this is a big question for such a small space. Let’s see how far we get. My wife is on the spectrum which makes being in a confined space with others quite difficult for her - and those around her. Confined space gives her little room for calming, so her anger events have increased.* Father of 1 child.

The burden of social isolation and keeping families healthy also weighed heavily on families dealing with physical illnesses, especially those involving compromised immunity. These families described the social isolation as “getting really hard now” and that they were “very fearful” given the high stakes involved in remaining in strict isolation.

*Our middle child is immunocompromised and at significant risk. We have been entirely isolating for 3 weeks now on medical advice. We do not leave our property, and all incoming goods are cleaned and sanitized.* Mother of 3 children.

Being housebound was also difficult for large families living in small houses without gardens or those in apartments, as space was a valued commodity. Parents used words such as “suffocating” and “going insane” to describe the experience of living in close quarters:

*All four of us are at home in a small townhouse so we lack quiet time. We’re juggling work, parenting, and home schooling constantly. It’s been stressful for the parents and a little boring for the kids.* Mother of 2 children.

Families facing economic worries also represented a group in need. As with the other themes, the impact of financial challenge appeared to exist along a continuum. Some families experienced “money worries,” while others faced serious deprivation, at odds with the image of Australia as a ‘land of plenty.’ Worries about being able to pay one’s mortgage or rent were common. Less common but extremely concerning were reports, such as from the following single mother, of having to forgo food to ensure that children had enough.

*Shopping alone is now a huge stress as I don’t want to expose my babies and the price rise in food has caused us now to only be able to buy enough food for a week so we are having less in each meal to ensure the children eat 3 meals a day. Most days I now miss meals so they can eat*. Mother of 2 children.

This quote illustrates the extreme economic deprivation endured by some families. Other common financial worries related to retaining work hours, depleting savings, or not being able to afford basics in the future. Some parents expressed concern that home-schooling and caring for children would cost them their job. Parents revealed that financial worries were a substantial part of their family’s deterioration in mental health:

*Massively. Financial stresses have affected the moods of the adults. Being home all the time has affected (greatly) the moods of the kids and adults. It’s really hard.* Mother of 3 children.

Being a single parent during the pandemic was also associated with substantial challenge, especially when dealing with the situation alone converged with other challenging life events:

*My husband left me on 02nd March. My job waa (sic) closed down at the end of March. It’s been a tough month. It’s been hard being home with 3 kids on my own. I’ve applied for single parent payment and still waiting for this to be finalized because centrelink is now so busy because of job seekers.* Mother of 3 children.

*Single parent, pregnant and have already had a meltdown. Struggling.* Mother of 2 children.

Other families that faced a difficult time were those enduring prejudice as a result of their backgrounds or working conditions. For example, medical workers expressed anxiety about prejudice from the general public in relation to spreading virus.

*I now do not wear my uniform other than to and from work for fear of abuse*. Mother of 2 children.

Another parent described racism.

*It has affected my mental health as I’m not comfortable going to the shops due to prejudice and the color of my skin.* Mother of 2 children.

Unfortunately, such prejudice was validated in a comment from another parent, who used a derogatory term to describe COVID19: *I’m worried about bringing the kung flu home to my family.*

It was clear that the pandemic was extremely burdensome to some families, but kinder to others. A number of families commented that their lives had little changed:

*As a full time stay at home mum, life is fairly normal for us. My husband has a secure job.* Mother of 1 child.

Families that retained access to employment, who described themselves as enjoying being at home, and as living in homes with expansive gardens and ample space seemed to report relatively few adverse effects. In fact, having access to protective factors, such as a large garden or strong family relationships, seemed to offset extensive burdens, as illustrated by the following parent, whose family had already experienced great difficulty emerging from the Australian bushfires:

*Well, this comes at the end of weeks of stress and worry due to the bushfire crisis and the impossibly high levels of pollution, having being caught up in the fires at the NSW South Coast, the freak hailstorm in Canberra, more fire and pollution (Orroral fire) and then this. Luckily we are a very close family, we enjoy each other’s company very much and we have a fairly large front and back gardens that allow plenty of different activities.* Father of 1 child.

#### Theme 6: Holding on to Positivity

Although an absolute sense of positivity was relatively rare, there were many examples of parents finding benefit, gratitude or ways to carry on despite the difficulties. Similar to the family relationships theme, there were some families who found opportunities for growth or intimacy in the face of challenge.

*I feel like the rug has been pulled out from under us, so far as my goals and plans for the next few years. But ultimately, we are doing well*. Mother of 1 child.

*I was quite panicked to begin with, but the kids love being with us all the time and are building their relationship with each other*. Mother of 2 children.

The pandemic provided an opportunity for the cultivation of positive qualities, including “appreciation”, “developing tolerance and understanding” as well as “learning to cope and develop patience.” Some parents expressed gratitude and a sense of relative fortunate: “We don’t feel as badly affected as some people”. Other sources of gratitude included having access to internet, a safe space to call home, enough food to eat, time to spend together, good health, financial stability and “having enough.” For some parents then, Australia remained the lucky country.

Parents also expressed gratitude about contextual factors, with one father commenting that the timing of restrictions likely contributed to their family’s positive response, but also, that the realities of home schooling were yet to be endured.

*We have adapted well, but are probably blessed by being in Victoria where school holidays occurred around the time of the strict social distancing measures came into effect. We have only just this week (three weeks after the strict measures) began schooling and kindergarten programs from home. I would anticipate that this new challenge will take quite a bit of getting used to.* Mother of 2 children.

Other families found benefit in the small, practical ways their lives had changed, and perhaps even improved. For example, less time spent commuting, with one father noting he saved 2.5 hours a day that he could now spend with his family, and another reporting less frenetic driving of children to their various activities.

*No more driving everywhere for tennis, piano, etc. Cross paths with wife & kids during the day!* Father of 3 children.

In fact, many fathers noted enjoyment in spending more time at home:

*I think it has helped improve work life balance, and I get to spend more time with the kids.* And *I spend more time with my children which is awesome.* Father of 2 children.

As picked up in the theme related to family relationships, a number of mothers confirmed that having their partner at home helped create opportunities for self-care and the opportunity to share home-care duties:

*My husband works from home now and I work night duty but it’s made our life a little better because I’m normally the one to have little sleep but my husband can take care of the kids why I sleep so I’m getting more sleep.* Mother of 5 children.

Lives were quieter and less hurried, and parents appreciated this slower pace, with one father noting: “in some ways life is busier but also calmer”, and a mother commenting “slowing things down to enjoy the day.” However, other families had mixed feelings: “We are less rushed, spending more time together. But there is underlying stress and anxiety,” as well as longing for the normal, “We enjoy not going anywhere but miss work and school.”

Parents commented on the activities or changes in their lives that made enduring the pandemic easier. Some families had grandparents move into the house to help care for children while parents were working from home. Others found solace in walking the dog, engaging in family activities together such as baking, crafts, and painting. Finding a new, sustainable routine, and keeping busy with enjoyable activities seemed to help families through the difficulties:

*Really struggled at the beginning due to social isolation. I was already a SAHM (mat leave) but we were very active (library, mother’s group, etc.). We had to adapt into a new schedule/routine, but have now settled in better than I thought we would.* Mother of 1 child.

*We mostly stay at home and play in the back or front yard. We also play games inside, cook and clean or tidy up mess. We have used the time stuck inside to rearrange the home and do a spring clean.* Mother of 3 children.

## Discussion

The findings from this exploratory qualitative study reveal novel insights into how the initial stages of the COVID-19 social restriction/isolation measures impacted Australian families, pointing to substantial mental health, parenting and relationship burdens. Although experiences were varied, with some families even flourishing under the restrictions, the predominant message expressed by families was hardship and loss.

Many parents described the significant emotional toll that the pandemic had on their own functioning, including experiencing increased levels of stress, and in one case, the contemplation of suicide. Parents also reported deteriorating mental health difficulties in their children, including the exacerbation of mental health difficulties in children with pre-existing mental health diagnoses, such as anxiety disorders and neurodevelopmental disorders. The unequal burden felt by parents of children with neurodevelopmental disorders is consistent with emerging research in the United Kingdom pointing to substantially higher stress levels for parents of children with special education needs or neurodevelopmental disorders (Waite et al., 2020). Parents reported a range of factors that may explain their mental health challenges, notably losing important connections to society—whether missing out on the playgrounds, libraries, and activities that provide interest and respite to families—or support from connecting with extended family or friends.

The various themes that emerged from participant responses together highlight how the stressors of the COVID-19 pandemic affected family relationships. For families with members who had pre-existing vulnerabilities, such as physical health concerns and mental health issues, the social restrictions and the economic downturn seemed to further tax their capacity to maintain positive relationship functioning and well-being. In contrast, the impact on relationships seemed to be less deleterious in families without pre-existing vulnerabilities and/or economic hardship. These findings align with widely studied models of relationship functioning and adaptation to stress (Hammen, 1991; Karney and Bradbury, 1995; Masarik and Conger, 2017), which predict worsening relationship outcomes as a function of higher levels of environmental stressors and adversity. In fact, high stress and adversity experienced at work by one family member can “spillover” to affect their functioning in their family roles, as parent and partner, and their stress, and mental health problems can also then “crossover” to affect other family members (Hammen, 1991; Repetti and Wang, 2017). If adaptive ways of managing relationships do not emerge during a time of financial hardship, then families can experience declines in relationship satisfaction (Masarik and Conger, 2017).

Parents reported that the COVID-19 pandemic had changed the nature and context of their parenting, and for many parents, it increased their experience of stress and reduced opportunities for support and respite, in some cases, placing unprecedented strain on parent-child relationships. Parents also expressed concerns about how the pandemic was affecting their child, such as worries that their child was missing out on education, healthcare, and on sports, social or other co-curricular activities. Our findings are consistent with research showing that environmental stressors, such as juggling paid work and stressful life events, are associated with more negative parenting outcomes, including lower parenting warmth, and increased parenting irritability (Conger et al., 2002, 2010). In the current study, many parents also discussed their difficulties in juggling childcare, or supporting their child’s remote learning, while working from home. Evidence shows that increases in parents’ experience of conflict in managing paid work and family responsibilities lead to strained family relationships, including more irritable parenting and couple conflict, with long-term negative impacts on child mental health outcomes (Dinh et al., 2017; Vahedi et al., 2018, 2019). It is not yet clear what the full impact of COVID-19 pandemic has been on parenting, but many parents reported facing unprecedented demands that they struggled to manage.

The impact of the pandemic was not uniform; some families faced considerably greater strain in relation to their context and the stressors that they were managing. Socioeconomic deprivation appeared to play a major role in determining the impact of the pandemic and a key source of the reported unequal burden among families. An unprecedented lack of resources due to COVID-19 related changes in employment, access to usual supports (both social and financial), and social isolation provided the precipitating milieu that likely resulted in a greater impact for some families already experiencing economic hardship. Of note, several recent COVID-19 related studies from around the world have also reported on the unequal burden of the pandemic, identifying high-risk populations, which have largely been characterized as experiencing high levels of poverty, comorbidities, and higher density living often involving smaller dwellings lacking outdoor space (Chin et al., 2020). Also of note, it is these same characteristics that may place an individual at greater risk for contracting COVID-19 (Chin et al., 2020).

Our results also reflect the possibility of benefit finding and meaning. Several parents noted they and their families experienced more adaptive and positive relationship functioning during lockdown via increased use of constructive communication and conflict resolution patterns when dealing with pandemic-related stressors. Others turned to each another or extended family for support to navigate competing responsibilities. These findings align with the Vulnerability Stress Adaptation Model (VSAM) of relationship adjustment (Karney and Bradbury, 1995) where adaptive relationship processes are thought to buffer the negative effects of stressors on relationship outcomes. Previous research on disasters also highlights individual differences in resilience, i.e., how people adapt in response to adversity (Rutter, 1985; Green, 2002; Preston, 2016; Southwick et al., 2016; Mendonca et al., 2019; Tiernan et al., 2019). In the present study, those who fared well during lockdown listed several protective factors, including employment security and financial stability, comfortable and safe homes, enough food to eat, good health, and also intrapersonal factors such as strong relationships. Moments of meaning—and even delight—including spending time with pets, and engaging in family activities together, assisted in families reframing the negative experience into an opportunity to develop appreciation, tolerance, patience, and learning. This cognitive reappraisal is a common psychological technique used in cognitive behavioral therapy, an effective psychotherapy for anxiety and depression (Cuijpers et al., 2013, 2014) and trauma (Kornør et al., 2008). Future studies should explore population-based interventions guiding people in cognitive reappraisal in prevention of post-pandemic mental illness. However, these approaches must be coordinated with more practical supports to families experiencing socio-economic hardship, since families are much less likely to benefit from therapeutic approaches when their basic needs are not met.

In our data, a number of fathers reported positive pandemic-related changes, including increased quality time spent with children. Within Australian culture, it is typical for fathers to spend relatively fewer hours providing care to children compared to mothers. The birth of a child often results in fathers working longer hours and mothers moving to part-time work to become the primary care-giver (Craig and Sawrikar, 2009). One unexpected, and perhaps positive outcome of the pandemic, is the opportunity for fathers to spend time at home, supporting their children and partners. It is important to further explore potential differences in the way fathers versus mothers have responded to the pandemic. For example, research has identified that women are more likely than men to report being burdened with family responsibilities during the pandemic, despite both men and women spending more time at home (ABS, 2020a), and being unemployed due to COVID-19 (ABS, 2020b). As such, women may be increasingly responsible for the difficult aspects of family life during the pandemic, including home schooling children, while fathers may be more likely to continue with employment, while having time to partake in the enjoyable aspects of family life. Further research is required to understand how COVD-19 has impacted gender roles. To mitigate the impact of the COVID-19 crisis on women, the Australian government proposed a series of strategies including increased support for women in pursuing employment opportunities, support for childcare, and promoting flexible work arrangements (Australian Government, 2020); however, it is unclear whether these measures will support mothers’ employability long term.

The present study had a number of notable strengths, including a large sample drawing on parents from a rich array of backgrounds, which supports the transferability of the findings to families across Australia. Limitations include the sole reliance on parents for data collection, such that the data are effectively “colored” by the parents’ account and perceptions. However, given the large sample, it was not feasible to collect data from children or others in the family to triangulate parent responses. In addition, the data reflect heterogeneous challenges that are dependent on the particular policies and timelines of states across Australia. For example, parents from Victoria were dealing with children home on Easter holidays, while parents from other states had already begun home schooling during this wave of data collection. Although all parents were facing restrictions and isolation, it is not possible to say that the pandemic was experienced by parents uniformly. The results need to be considered in light of the likelihood that additional challenges were still to come for families not yet home-schooling, and responses may continue to shift and emerge across the pandemic.

Our findings show that for many families, the pandemic has presented an exacerbation of previous mental health problems or the presentation of new mental health problems. Given that parent mental health problems are associated with a broad range of downstream effects, such as increased relationship conflict, irritable parenting (Vahedi et al., 2019), increased difficulties in juggling work and family responsibilities (Westrupp et al., 2015), and co-morbid health problems (Ohrnberger et al., 2017), it is imperative that parents seek help for any persistent and impairing family mental health difficulties. Currently in Australia, we have strong public health messaging about the need to be tested for coronavirus, but arguably of importance too, is public health messaging about when to seek mental health support and how to access services. A number of phone support and web chat support services have been established to provide information, advice, and strategies to assist with mental health during the pandemic in Australia. We have also seen the roll out of telehealth services in Australia, which can be accessed via rebated sessions. However, the decision to use such services requires individual knowledge and judgment about when to reach out for help. Given the extent of mental health and other challenges reported herein, many families are likely to need ongoing support.

## Data Availability Statement

The data will be available for participants who consented to their de-identified information being shared for use in ethically approved and related studies.

## Ethics Statement

The studies involving human participants were reviewed and approved by the Deakin University Human Ethics Advisory Group (Project number: HEAG-H 52_2020). Written informed consent for participation was not required for this study in accordance with the national legislation and the institutional requirements.

## Author Contributions

SE, EW, and AM-W were involved in the study design, execution, data analysis, and write up. AK, LO, ES, and GK were involved in the data analysis and write up. All authors contributed to the article and approved the submitted version.

## Conflict of Interest

The authors declare that the research was conducted in the absence of any commercial or financial relationships that could be construed as a potential conflict of interest.
